# Electro-acupuncture for irritable bowel syndrome patients: study protocol for a single-blinded randomized sham-controlled clinical trial

**DOI:** 10.1186/s13063-021-05563-4

**Published:** 2021-09-15

**Authors:** Linda L. D. Zhong, Tsz Fung Lam, Wei Yang, Ya Zheng, Zipan Lyu, Zhaoxiang Bian

**Affiliations:** 1 Hong Kong Chinese Medicine Clinical Study Centre, Hong Kong Baptist University, 3F, Jockey Club Chinese Medicine Building, 7 Baptist University Road, Kowloon, Hong Kong, SAR P. R. China; 2 School of Chinese Medicine, Hong Kong Baptist University, Jockey Club Chinese Medicine Building, 7 Baptist University Road, Kowloon, Hong Kong, SAR P. R. China

**Keywords:** Irritable bowel syndrome, IBS, Electro-acupuncture

## Abstract

**Background:**

Irritable bowel syndrome (IBS) is one of the most common functional gastrointestinal disorders in clinical practice. IBS diagnosis is based on symptoms defined by abdominal pain or discomfort associated with defecation or changes in bowel habits. Gut-brain interaction caused by stress or depressive emotion is one of the essential pathologies. Acupuncture has been used for the treatment of internal medicine, including digestive disorders and depressive disorders in Chinese medicine. This study aims to determine whether electro-acupuncture could have significant benefits than sham acupuncture for IBS.

**Methods/design:**

This is a single-blinded randomized sham-controlled clinical trial with two arms. A total of 120 IBS patients will be recruited. After a 2-week run-in period, eligible subjects will be randomly assigned to one of two arms, acupuncture (AC) arm and sham acupuncture (SAC) arm. Each eligible subject will go through a 2-week run-in-period, 6-week treatment period, and 6-week follow-up period. Five visits in total were scheduled for each subject in week 0, week 2, week 5, week 8, and week 14. The outcomes would be measured with (1) IBS-SSS, (2) Hamilton Depression Rating Scale (HAMD-17), (3) Clinical Global Impression-Severity (CGI-S), (4) Self-Rating Depression Scale (SDS), and (5) IBS Quality of Life (IBS-QoL).

**Discussion:**

The study will compare electro-acupuncture with sham acupuncture to explore the feasibility of electro-acupuncture in improving IBS symptoms.

**Trial registration:**

ClinicalTrials.govNCT04387383. Registered on 13 May 2020

**Supplementary Information:**

The online version contains supplementary material available at 10.1186/s13063-021-05563-4.

## Background

Irritable bowel syndrome (IBS) is one of the most common functional gastrointestinal disorders in clinical practice. The diagnosis of IBS is based on symptoms defined by abdominal pain or discomfort associated with defecation or changes in bowel habits [1]. Recent pooled population-based meta-analysis indicated that the global prevalence of IBS was approximately 11.2% [2], and an early epidemiological study in Hong Kong showed that 13% of males and 21% of females in Hong Kong suffered from IBS [3]. To date, the treatment for IBS is unsatisfactory. Moreover, people with IBS frequently suffer from anxiety and depression, which can worsen the symptoms. Of those who do seek treatment, research has found that 54 to 94% have a psychiatric disorder such as an anxiety disorder or depression [4–6]. Up to the present, there has not been a satisfactory treatment of IBS in pharmacological approach nor complementary medicine [7]. In China, antibiotics, antispasmodics, and secretagogues are used to treat IBS, but the effectiveness varies [8]. Non-medication treatments such as dietary/nutritional and psychobehavioral therapy can be the alternatives; however, the requirement of high adherence may decrease the rates of treatment continuation [9, 10]. Acupuncture can be a more convenient option for patients with IBS as the treatment does not require daily intake of medication nor frequent clinical visits.

Acupuncture has been practiced empirically in China for several millennia, and it is increasingly accepted by practitioners and patients worldwide, especially during the last three decades [11, 12]. Currently, acupuncture treatment for IBS is a research hotspot in alternative medicine. A trial of acupuncture in 233 patients with IBS published in 2012 showed acupuncture improved their quality-adjusted life years [13]. Other reviews indicated that acupuncture was effective for IBS global symptoms [14, 15]. Research showed that electro-acupuncture had the same effectiveness as medication in decreasing diarrhea on IBS patients with additional improvement on the consistency of stool and visceral pain symptoms [16]. Besides, it is well demonstrated that acupuncture has broad therapeutic benefits in treating various psychiatric disorders. A recent meta-analysis indicated that acupuncture treatment was effective in alleviating various depressive symptoms [17].

A cohort study concluded that IBS patients had an increased risk of developing depressive disorder [18]. At the same time, the disorder is also a risk factor of IBS; therefore, the intervention on depression is critically important. Among the previous researches, abdominal points are most frequently selected and used as core points for IBS treatment. However, these points mainly relieve IBS local symptoms such as bloating and visceral pain [19–22]. Scalp points can be used in combination with the abdominal ones to treat global symptoms like headache, fatigue, or mood change. We may infer that scalp-abdominal point acupuncture is an effective treatment option for overall symptoms of IBS. However, there is no available clinical research applying multiple scalp points in the treatment nor demonstrating this conclusion [23]. Thus, we designed this trial with scalp-abdominal acupoints to be the fixed and core points and hypothesized that acupuncture could produce more remarkable clinical improvement compared to sham acupuncture for IBS symptoms.

## Objectives

To test the hypothesis, a single-blinded, randomized, sham-controlled trial is designed to determine whether acupuncture could have significant benefits than sham acupuncture for IBS.

## Materials and methods

### Study design

**Fig. 1:**
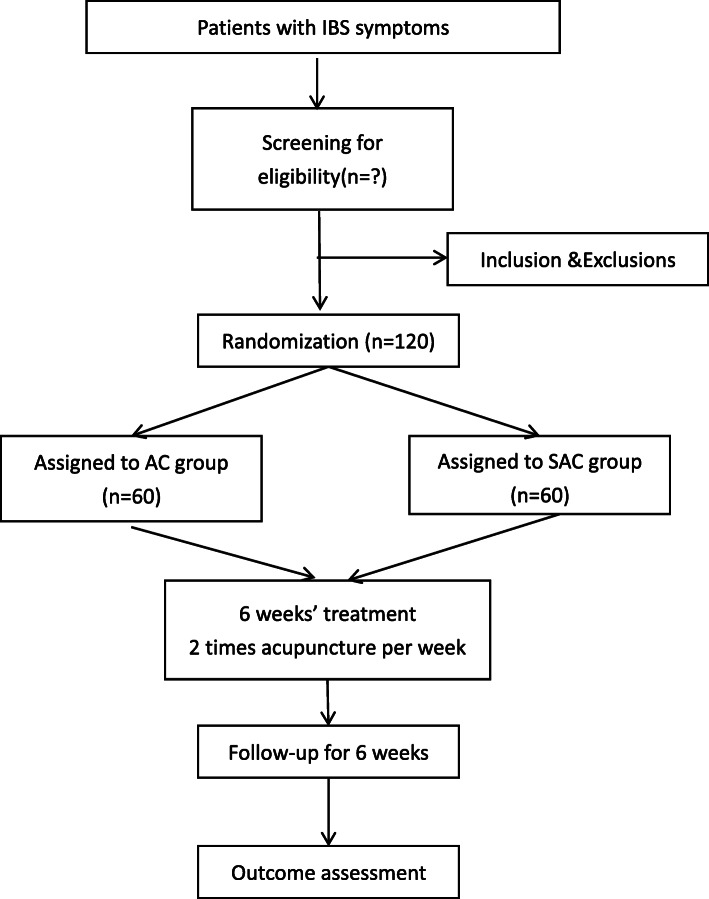
Participants flow chart

**Fig. 2:**
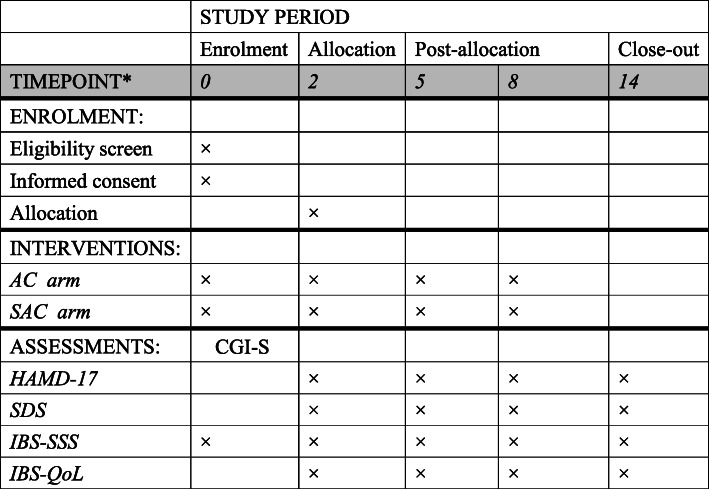
Participant timeline

This is a single-blinded randomized sham-controlled clinical trial with two arms. A total of 120 IBS patients will be recruited. The study will cooperate with Hong Kong Baptist University and University of Toronto. After a 2-week run-in period, eligible subjects will be randomly assigned to one of two arms, acupuncture (AC) arm and sham acupuncture (SAC) arm. Each eligible subject will go through a 2-week run-in-period, 6-week treatment period, and followed by a 6-week follow-up period. Five visits in total were scheduled for each subject in week 0, week 2, week 5, week 8, and week 14. The participant flowchart is listed in Fig. 1, and the participant timeline is listed in Fig. 2.

### Participants

#### Diagnostic criteria for IBS (Rome IV) [24]

Recurrent abdominal pain, on average, at least 1 day per week in the last 3 months with symptom onset at least 6 months, associated with two or more of the following criteria: (1) related to defecation, (2) associated with a change in frequency of stool, and (3) associated with a change in form (appearance) of stool.

#### Classification of subtypes

After diagnosis, participants will be given a questionnaire with the Bristol Stool Form Scale (BSFS) to classify their subtypes. The study includes all IBS subtypes (Additional file 7).

### Inclusion criteria

Patients will be included if they have all the following at baseline and during the 2-week run-in period:

1. Fulfillment of the Rome IV criteria for IBS
2. Age of 18 to 65 years (inclusive)
3. IBS Symptom Severity Scale (IBS-SSS) (Appendix 1) > 75 points (a range of 0–500 points of VAS on five questions)

### Exclusion criteria

Patients will be excluded if they have one or more of the following:

1. Pregnancy or breast-feeding
2. Medical history of inflammatory bowel diseases, carbohydrate malabsorption, hormonal disorder, known allergies to food additives, and/or any other serious diseases
3. Unstable medical conditions
4. Unstable mental condition or with a history of mental illness
5. Patients who have received acupuncture treatment in the last 3 months or took concomitant medication with effects of gastrointestinal motility or visceral sensation, such as antidiarrheal agent, antidepressant, narcotic analgesic, and anticholinergic
6. Alcoholism or drug abuse in the past 1 year
7. Having needle phobia

#### Withdraw from the trial

Participants will be allowed or asked to drop out from the trial if they:

1. Are lost to follow-up
2. Become pregnant
3. Develop serious adverse event (SAE)

Participants can withdraw from this clinical trial at any time. The date and reason for withdrawal should be stated. If possible, all subjects withdrawing from the study should continue to be followed up regularly on a measurement schedule with a final assessment. Participants who discontinue early will not be replaced.

### Recruitment

We will recruit participants through advertisements in newspapers and TV programs. Screening will be done by researchers. Informed consent will be obtained from eligible patients before randomization.

### Intervention

Each patient will be scheduled for a total of 12 treatment sessions, 30 min for each session, two times a week over a 6-week period. The selection of acupuncture points is based on the traditional Chinese medicine (TCM) theory [24–26] and evidence-based clinical research [22]. According to the traditional Chinese medicine (TCM) theory, the spleen transforms the food digested by the stomach into essences. It helps to transport throughout the body, while the liver ensures the smooth flow of Qi, modifies the activity of internal organs (Zang Fu), and is highly related to the emoto-psychological stage. Therefore, most TCM experts agree that the weakness of the spleen and stomach is the basic pathogenesis of IBS, while the stagnant among the liver and spleen can also cause a serious symptom of IBS [27]. Therefore, the principles of treatment of IBS are regulating the Qi movement among the liver and spleen. Thus, the following acupuncture points are selected for the treatment (Tables 1 and 2).

**Table 1:** Checklist for items in STRICTA 2010

Item	Detail
**1. Acupuncture rationale**	**1a) Style of acupuncture** Manual and electro-acupuncture base on traditional Chinese medicine theory and evidences from previous clinical trials.
	**1b) Reasoning for treatment provided, based on historical context, literature sources, and/or consensus methods, with references where appropriate** According to systematic reviews and clinical experiences of our principal investigator and co-investigators.
	**1c) Extent to which treatment was varied** Standard treatment is used. No variation of treatment among patients.
**2. Details of needling**	**2a) Number of needle insertions per subject per session (mean and range where relevant)** 15 needles.
	**2b) Names (or location if no standard name) of points used (uni/bilateral)** Bilateral—Tianshu (ST25), Zusanli (ST36), Sanyinjiao (SP6), Toulinqi (GB15), Taichong (LR3), and Zhangmen (LR13). Unilateral—Baihui (GV20), Guanyuan (CV4), and Zhongwan (CV12).
	**2c) Depth of insertion, based on a specified unit of measurement, or on a particular tissue level** 10–30 mm.
	**2d) Response sought (e.g.,*de qi*or muscle twitch response)** De qi.
	**2e) Needle stimulation (e.g., manual, electrical)** Manual. Electrical—dense-disperse waves with 50 Hz at 10 volts.
	**2f) Needle retention time** 30 min.
	**2g) Needle type (diameter, length, and manufacturer or material)** Disposable stainless steel acupuncture needles (Dong Bang acupuncture needles, 0.25 mm in diameter and 40 mm in length with guide tube, manufactured by Dong Bang Acupuncture Inc. Korea).
**3. Treatment regimen**	**3a) Number of treatment sessions** 12 sessions.
	**3b) Frequency and duration of treatment sessions** 2/week for 6 consecutive weeks.
**4. Other components of treatment**	**4a) Details of other interventions administered to the acupuncture group (e.g., moxibustion, cupping, herbs, exercises, lifestyle advice)** None.
	**4b) Setting and context of treatment, including instructions to practitioners and information and explanations to patients** University clinics. Participants will be informed about acupuncture treatment in the study as follows: “In this study, acupoints for IBS will be used based on related reports and clinical experience of our investigators.”
**5. Practitioner background**	**5) Description of participating acupuncturists (qualification or professional affiliation, years in acupuncture practice, other relevant experiences)** Hong Kong-registered Chinese medicine practitioners having at least 3 years of clinical experience, who have undergone training and are able to provide identical acupuncture treatment in accordance with a pre-defined protocol.
**6. Control or comparator interventions**	**6a) Rationale for the control or comparator in the context of the research question, with sources that justify this choice** To assess the efficacy and safety of electro-acupuncture compared to sham acupuncture.
	**6b) Precise description of the control or comparator. If sham acupuncture or any other type of acupuncture-like control is used, provide details as for items 1 to 3 above** - Style of acupuncture: sham acupuncture (needling at sham acupoints). - Number of needle insertions per subject per session: 15 needles. - Depth of insertion: 10–30 mm. - Needle retention time: 30 min. - Needle type: the same as the treatment group. - Number of treatment sessions: 12 sessions. - Frequency and duration of treatment sessions: 2/week for 6 consecutive weeks.

*Note*: This checklist, which should be read in conjunction with the explanations of the STRICTA items, is designed to replace CONSORT 2010’s item 5 when reporting an acupuncture trial

**Table 2:** Acupuncture points

Acupuncture point	Anatomical location	Function according to TCM
Baihui (GV20)	7 cun directly above the midpoint of the posterior hairline	Easing mental stress
Toulinqi (GB15)	Directly above the pupils when the eyes are looking straight ahead, 0.5 cun within the anterior hairline, at the midpoint of the line connecting Shenting (GV24) and Touwei (ST8)	Tranquilizing the mind
Guanyuan (CV4)	On the anterior midline, 3 cun below the umbilicus	Regulating qi
Zhongwan (CV12)	On the anterior midline, 4 cun above the umbilicus	Regulating the stomach to smooth qi
Tianshu (ST25)	2 cun lateral to the umbilicus	Regulating qi flow for activating stagnancy
Zusanli (ST36)	3 cun below Dubi (ST35), one finger width lateral to the anterior crest of the tibia	Strengthening the spleen and stomach
Sanyinjiao (SP6)	3 cun above the medial malleolus, on the posterior border of the medial aspect of the tibia	Strengthening the spleen and stomach
Taichong (LR3)	In the depression distal to the junction of the 1st and 2nd metatarsal bones	Calming down the liver and stop the pain
Zhangmen (LR13)	On the lower border of the free end of the 11th rib	Spreading the liver qi and stop the pain

### Acupuncture (AC) group

Disposable acupuncture needles (0.30 mm in diameter and 25–40 mm in length) are inserted at a depth of 10–30 mm obliquely into the scalp acupuncture points (Baihui, Toulinqi) or straightly into the body acupuncture points (Guanyuan, Zhongwan, Tianshu, Zusanli, Sanyinjiao, Taichong, Zhangmen). Electro-acupuncture will be applied to the abdominal points at fast and dispersed waves through an electric needle stimulator (ES-160 6-Channel Programmable Electro-acupuncture) for 30 min. The intensity is adjusted to a level at which patients feel comfortable. The alternating stimulation is believed to produce maximal biochemical responses in the brain [28].

### Sham acupuncture (SAC) group

**Fig. 3:**
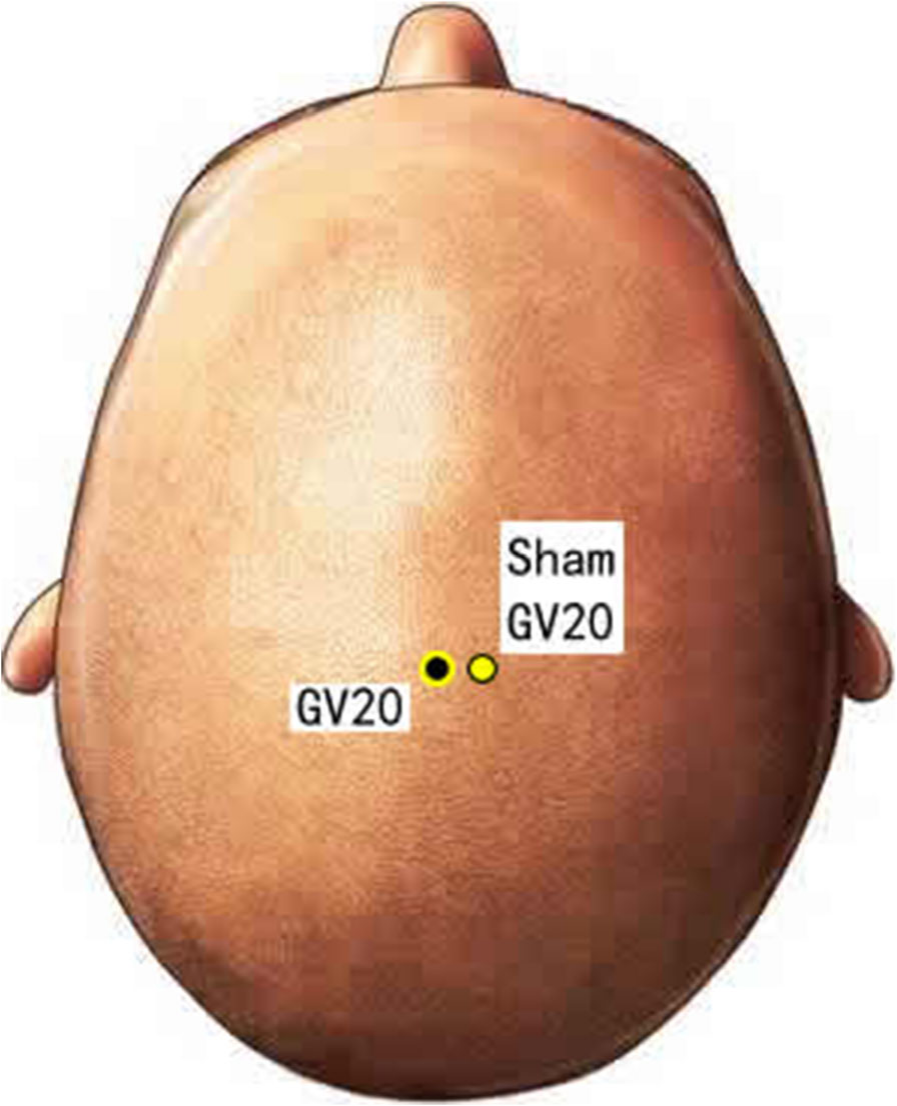
Sham points: GV 20 and Sham GV20

**Fig. 4:**
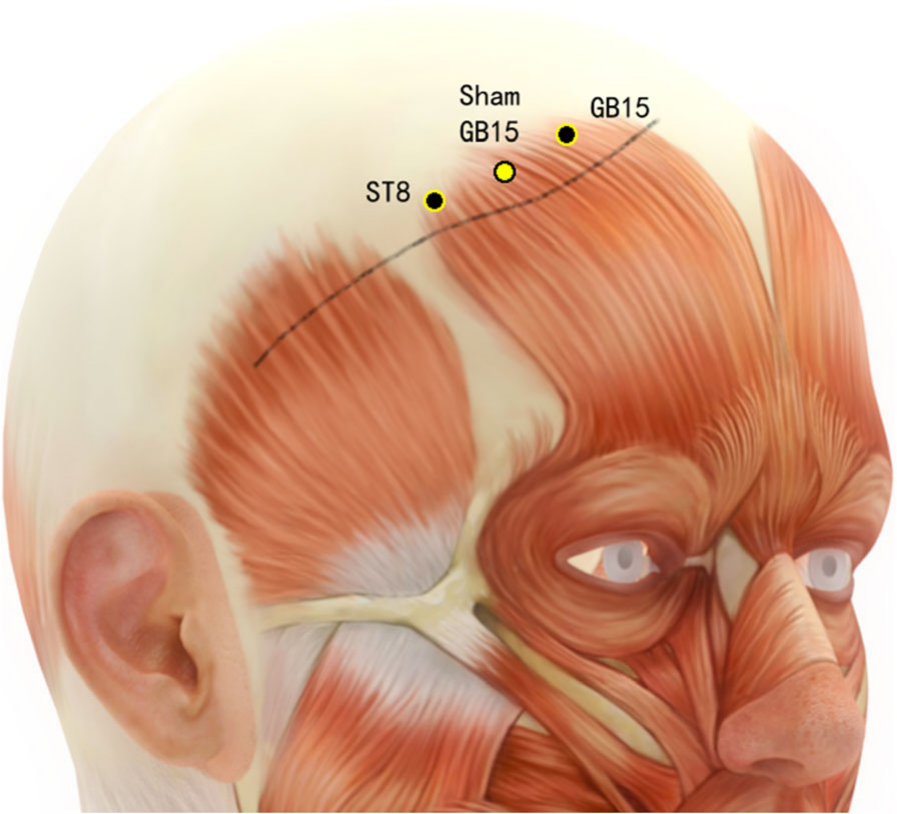
Sham points: ST8, Sham GB15, and G15

**Fig. 5:**
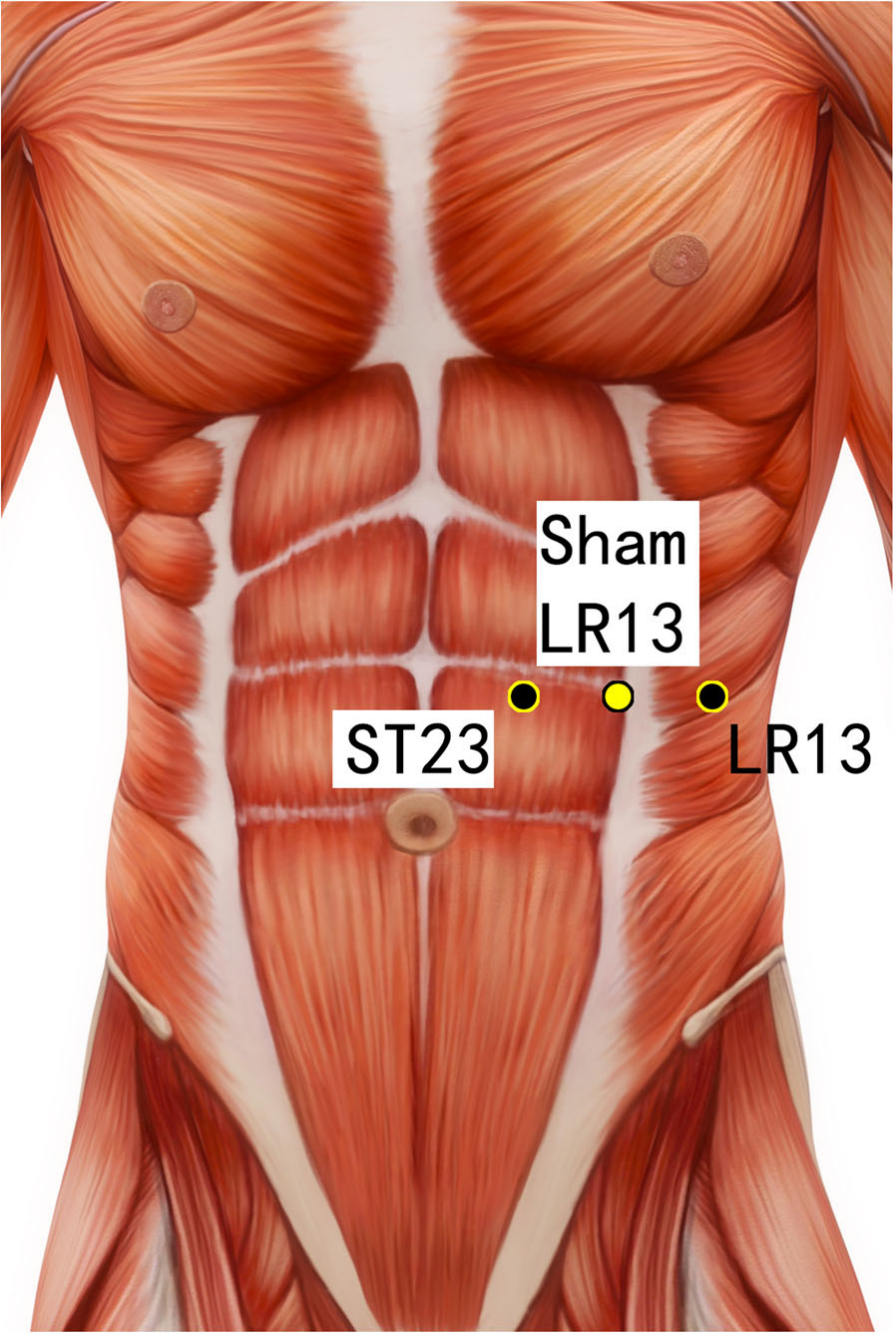
Sham points: ST23, Sham LR13, and LR13

**Fig. 6:**
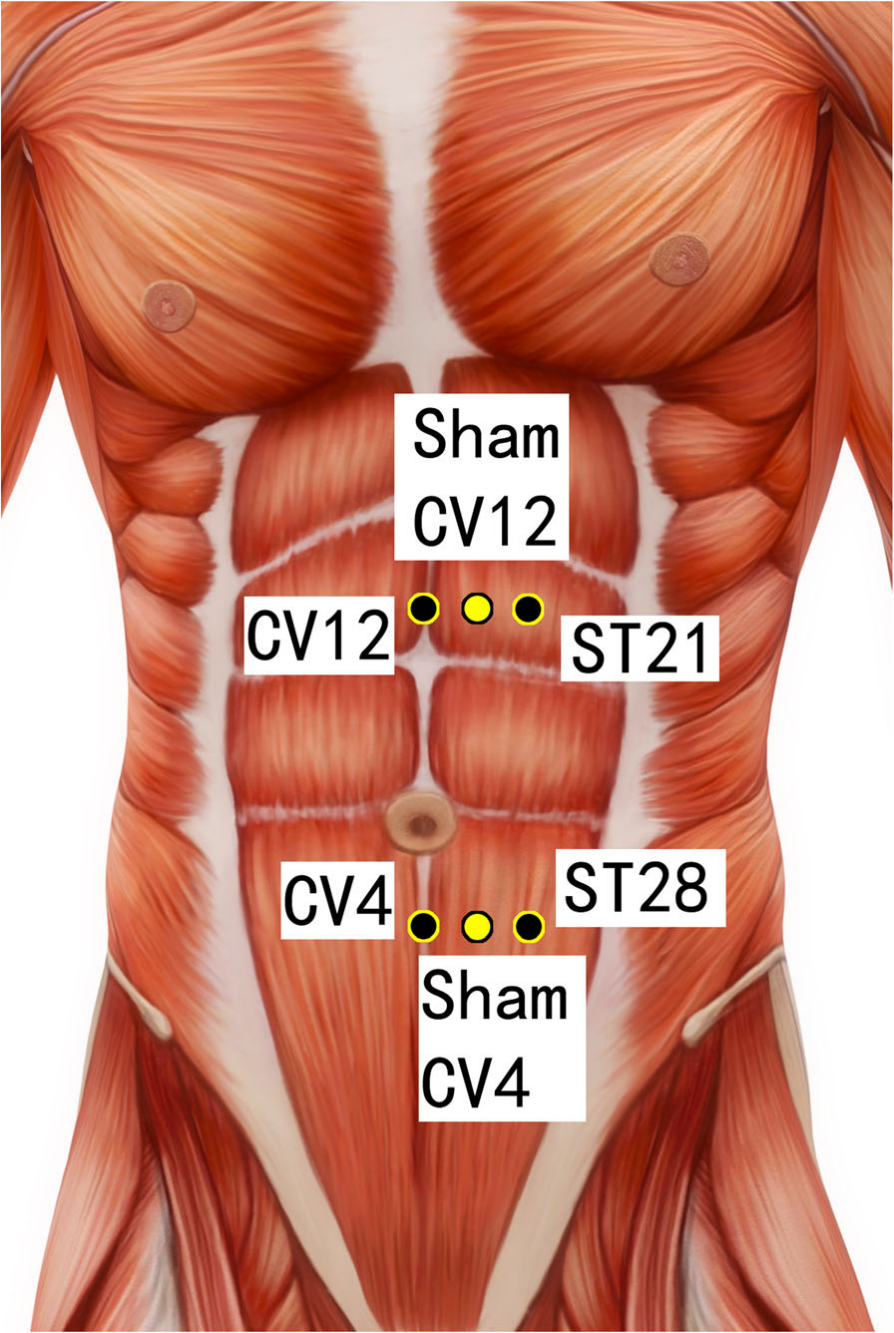
Sham points: CV12, Sham CV12, ST21, CV4, Sham CV4, and ST28

**Fig. 7:**
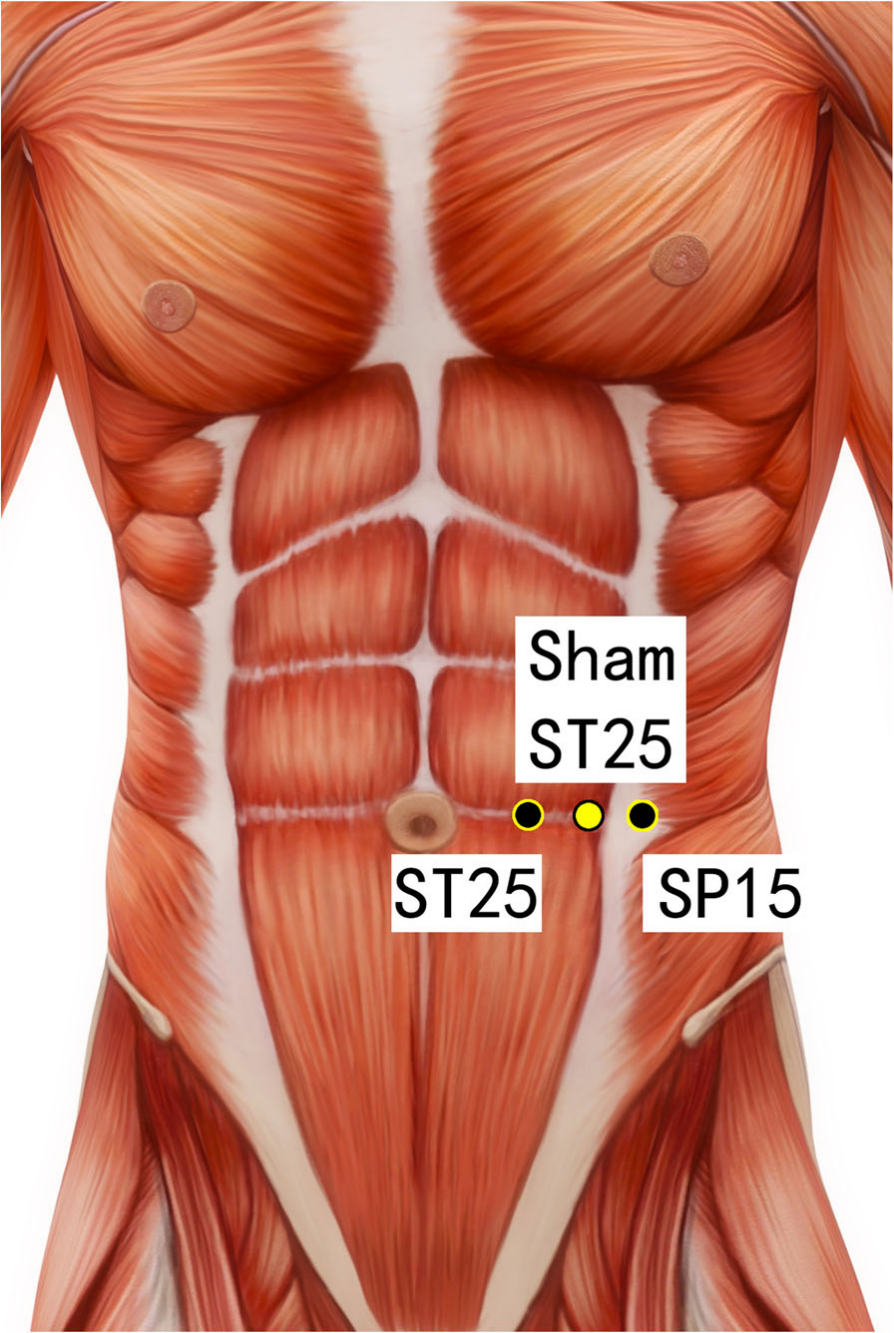
Sham points: ST25, Sham ST25, and SP15

**Fig. 8:**
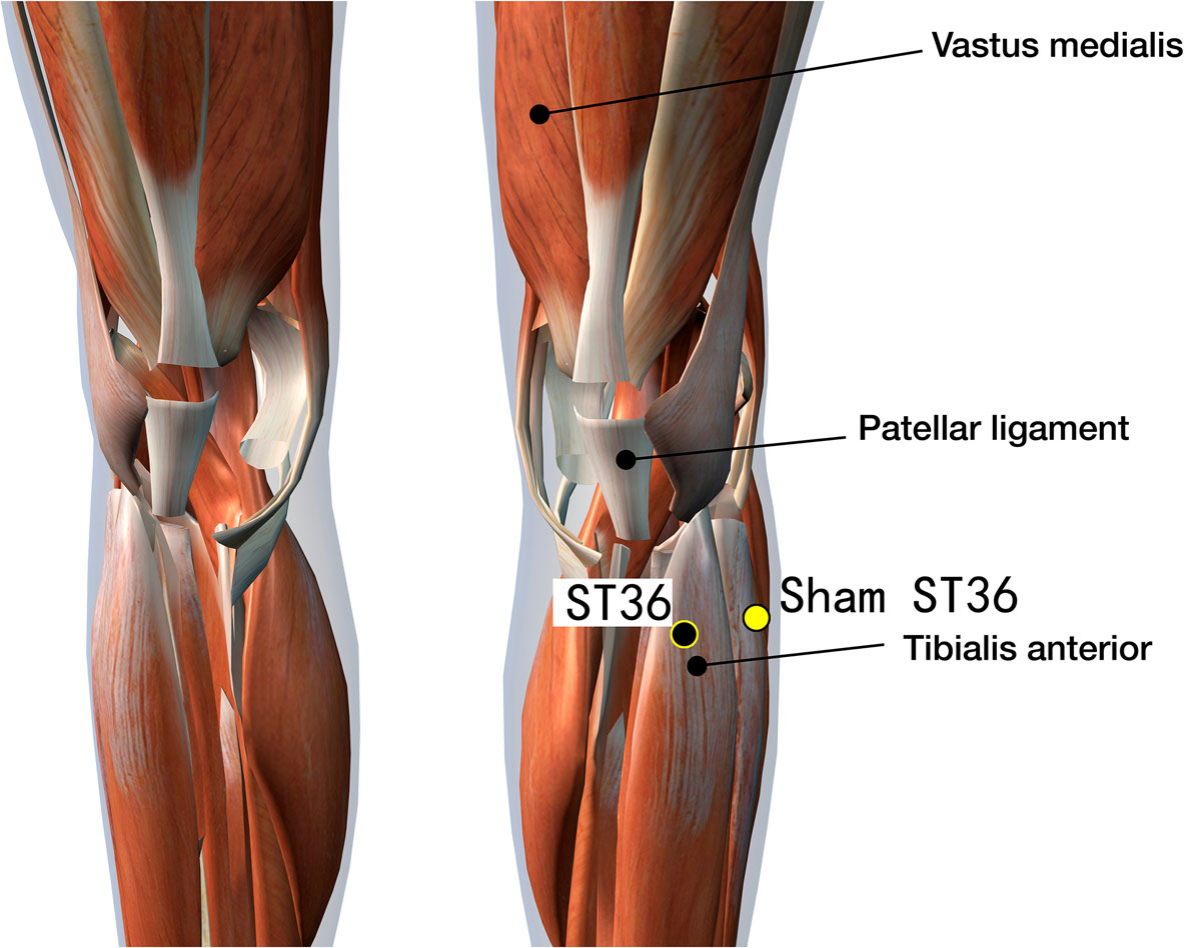
Sham points: ST36 and Sham ST36

**Fig. 9:**
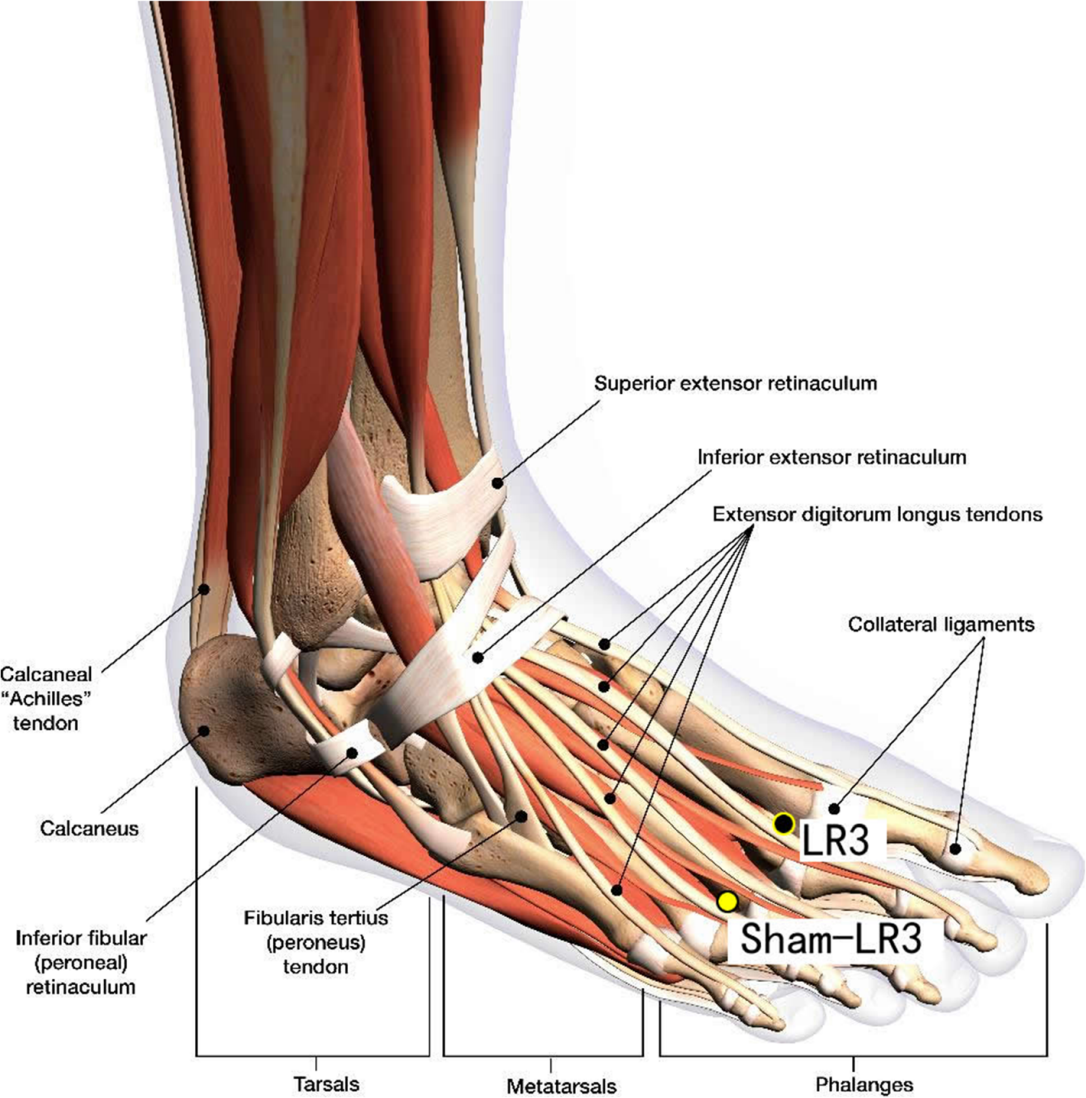
Sham points: LR3 and Sham LR3

**Fig. 10:**
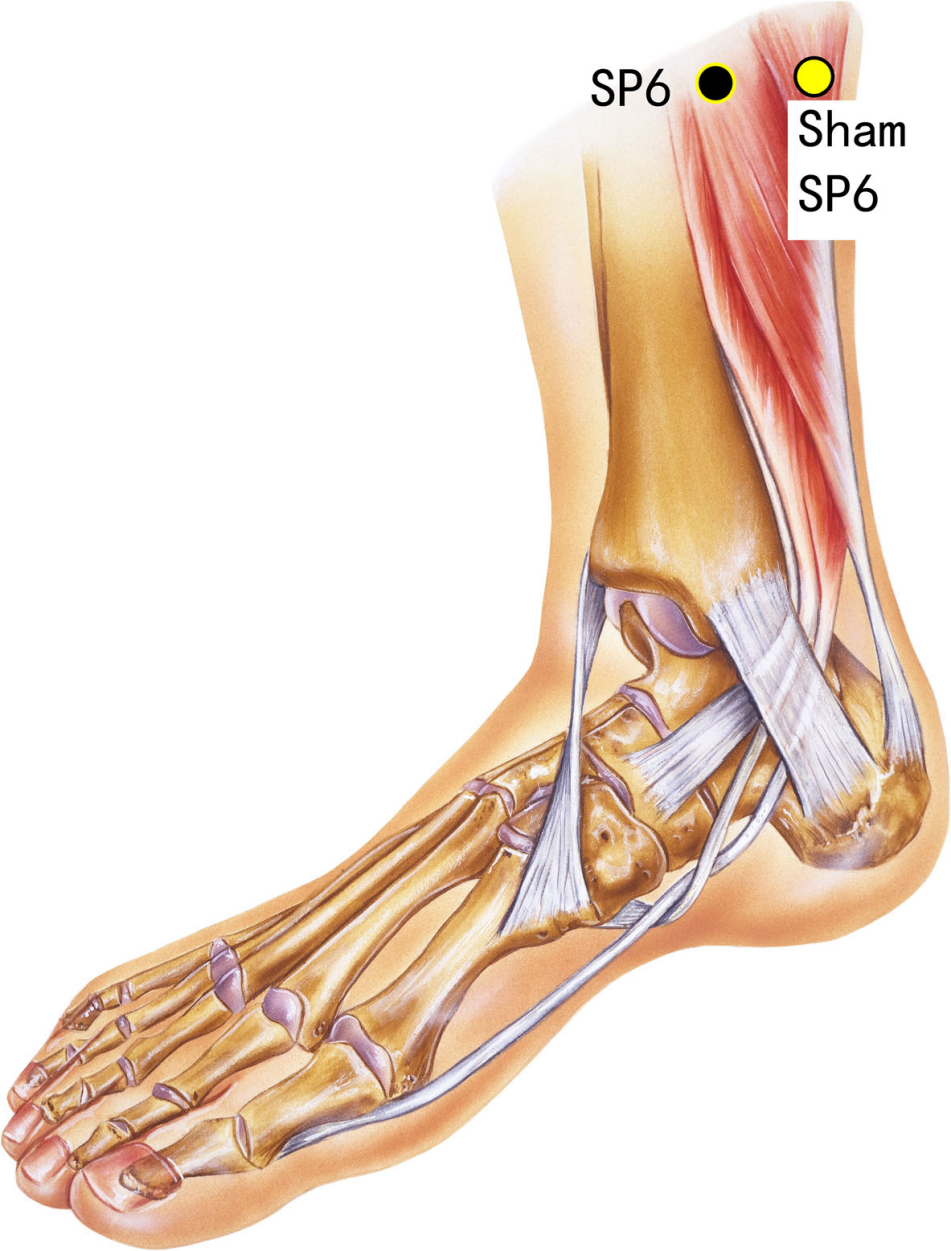
Sham points: SP6 and Sham SP6

Disposable acupuncture needles (0.30 mm in diameter and 25–40 mm in length) are inserted at the same way as in the acupuncture group but on the sham acupuncture points (Sham-Baihui, Sham-Toulinqi, Sham-Guanyuan, Sham-Zhongwan, Sham-Tianshu, Sham-Zusanli, Sham-Sanyinjiao, Sham-Taichong, Sham-Zhangmen; Table 3, Figs. 3, 4, 5, 6, 7, 8, 9, and 10). The sham points are non-acupuncture points nor located on the meridians [29].

**Table 3:** Sham acupuncture points

Sham points	Location
Sham-Baihui (GV20)	0.5 cun lateral from GV20
Sham-Toulinqi (GB15)	At the midpoint of the line connecting GB15 and ST8 (0.5 cun above the hairline, at the corner of the forehead, 4.5 cun lateral from the midline), 0.5 cun within the anterior hairline
Sham-Guanyuan (CV4)	In the mid-way of CV4 and ST28, 1 cun lateral to the anterior midline, 3 cun below the umbilicus
Sham-Zhongwan (CV12)	In the mid-way of CV12 and ST21, 1 cun lateral to the anterior midline, 4 cun above the umbilicus
Sham-Tianshu (ST25)	In the mid-way of ST25 and SP15, 3 cun lateral to the umbilicus
Sham-Zusanli (ST36)	Horizontal to ST36, on the edge of the fibula
Sham-Sanyinjiao (SP6)	Horizontal to SP6, 3 cun above the depression between the tip of the medial malleolus and the tendo calcaneus (KI3)
Sham-Taichong (LR3)	In the depression distal to the junction of the 3rd and 4th metatarsal bones, between the stomach and gall bladder channel
Sham-Zhangmen (LR13)	In the mid-way of LR13 and ST23 horizontally, 2 cun above the umbilicus, on the outer edge of the rectus abdomius

### Outcomes

The primary outcome is the difference between baseline and 8 weeks and 14 weeks after randomization on IBS Symptom Severity Scale (IBS-SSS).

The secondary outcomes include (1) the difference between baseline and 8 weeks and 14 weeks after randomization on Hamilton Depression Rating Scale (HAMD-17), (2) the difference between baseline and 8 weeks and 14 weeks after randomization on Clinical Global Impression-Severity (CGI-S) and Self-Rating Depression Scale (SDS), and (3) the difference between baseline and 8 weeks and 14 weeks after randomization on IBS Quality of Life (IBS-QoL). IBS-SSS has been validated as a classical monitor of the disease since 1997 and has been widely adopted across IBS researches [30]. The specific assessment schedule is in Fig. 2. Safety profiles will be assessed by determining the important adverse events reported on in every treatment and follow-up interviews.

### Assignment and blinding

Simple, complete, non-sequential randomization numbers will be generated by the Random Allocation Software (Version 1.0.0), Isfahan, Iran, and kept by the principal investigator (PI). After the patient’s eligibility is confirmed, patients will be randomized to AC arm or SAC arm for 6 weeks. Only the acupuncturists are allowed to know the patients’ treatment. All other research members will be blind to the assignment. In addition, patients will also be blinded. They cannot visually detect sham or active acupuncture procedures.

### Sample size calculation

According to a previous study, placebo is associated with high rates of resolution in a functional bowel disorder. Assuming that a difference of at least 30% between acupuncture and placebo is needed for a clinically important outcome, 51 patients per treatment group were deemed sufficient to achieve 80% power in detecting a treatment difference, based on a two-sided *χ*^2^ test without continuity correction at a significance level of 0.025 (used to maintain the overall significance level at 5%). Further assuming a 15% dropout rate, we concluded that we needed to recruit a total of 120 patients (60 per arm) for this trial to ensure statistically significant results. The calculation was performed using the StudySize 3.0 software (V. Frolunda, Sweden).

### Statistical analysis

All efficacy and safety analyses will be conducted according to the intention-to-treat (ITT) principle. Missing values will be imputed by the last-observation-carried forward method. The statistical analysis will be performed using the Statistical Packages of Social Sciences (SPSS) for Windows version 25.0. The statistical significance is defined as a two-sided *P*-value of < 0.05. Baseline differences between the groups will be assessed with the use of Student’s *t*-test for normally distributed continuous variables and the non-parametric Mann-Whitney *U* test for non-normally distributed. For categorical variables, the chi-squared test or Fisher’s exact test will be used. Changes in IBS-SSS, HAMD-17, SDS, and IBS-QoL at each evaluation time point from baseline will be analyzed using the ANCOVA model, followed by the Bonferroni *t*-test to detect the differences between the two groups at each time point. Incidence of adverse events will be examined using the chi-square test.

## Discussion and conclusion

The study is a single-blinded, randomized controlled clinical trial to evaluate the efficacy and safety of acupuncture for IBS, including all subtypes: IBS-C, IBS-D, IBS-M, and IBS-U. In addition to the change of bowel habits, people with IBS frequently suffered from anxiety, and depression or mood change further worsens the IBS. Currently, there is not any study on acupuncture in treating IBS digestive-psychological symptoms; therefore, the scalp points and abdominal points are included in this clinical trial. It would be the first of such research.

If the outcome from this study shows improvement in IBS, we may expect a larger scale of study. Research on the integration of non-medication treatments can be developed with the data from this clinical trial, such as electro-acupuncture with diet or lifestyle change, or integration of acupuncture and psychological cognitive-behavioral therapy.

The limitation of this study is that the selection of acupuncture points is standardized and utilized for every subject without syndrome differentiation. IBS can be differentiated into 4 subtypes, and each can be further sub-divided with Chinese medicine syndrome differentiation such as spleen dysfunction, liver depression, kidney Yang deficiency, and intestinal heat dampness. Although dysfunction of the spleen system is the leading cause of the problem, point selection according to each subtype may optimize the effectiveness of acupuncture. In addition, the environmental factors and dietary factors of the participants exposed to may affect the study result. We would require participants to record their daily diet on a diet diary, but no dietary modification would be given in our study.

In conclusion, this study will provide the basis for the effectiveness and safety of electro-acupuncture on IBS global treatment and would explore the possibility of acupuncture in the integrative treatment of psychological conditions.

## Trial status

A pilot study with a sample size of 20 (14.3% of the total 140 participants, 10 in each arm) has been done to estimate the efficacy of this protocol and to observe the outcomes and any development of adverse events. The rate of recruitment for the pilot trial of 20 participants is 100%. Another 120 participants will be recruited from August 2021. The study will be completed in July 2022. The schedule may be delayed with the COVID-19 pandemic which may be discouraging for patients joining any clinical trials. The problem would be mitigated with our fully vaccinated clinical staff and the growing vaccination rate in the Hong Kong population.

## Supplementary Information

**Additional file 1.** IBS-SSS.

**Additional file 2.** HAMD-17.

**Additional file 3.** CGI-S.

**Additional file 4.** SDS.

**Additional file 5.** IBS-QoL.

**Additional file 6.** Consent form.

**Additional file 7.** Questionnaire with the Bristol Stool Form Scale.

## Data Availability

The datasets used and analyzed during the current study are available from the corresponding author on reasonable request.
