# Supplementary material for: Electro-acupuncture for irritable bowel syndrome patients: study protocol for a single-blinded randomized sham-controlled clinical trial
Source: Trials. 2021 Sep 15;22:619. doi: 10.1186/s13063-021-05563-4 (PMC8441043; doi:10.1186/s13063-021-05563-4)
Supplement: Supplementary file 1 — Additional file 1. IBS-SSS. [file 13063_2021_5563_MOESM1_ESM.docx]

**IBS-SSS**

1. SCORE

a) Do you currently suffer from abdominal (tummy) pain? YES/NO

b) If yes, how severe is your abdominal (pain)?

0%

100%

No pain

Very severe

Quite severe

Not very severe

severe

c) Please enter the number of days that you get the pain in every ten days.

Number of days with pain: x 10

2.

a) Do you currently suffer from abdominal distension*? YESNO

(bloating, swollen or tight tummy)

(*women, please ignore distension related to your periods)

b) If yes, how severe is your abdominal distension/tightness

0%

100%

No distension

Very severe

Quite severe

Not very severe

severe

3. How satisfied are you with your bowel habit?

0%

100%

Very happy

Very unhappy

Quite happy

Unhappy

4. Please indicate with a cross on the line below how much your Irritable

Bowel Syndrome is affecting or interfering with your life in general

0%

100%

Not at all

Completely

Not much

Quite a lot
