# Supplementary material for: Electro-acupuncture for irritable bowel syndrome patients: study protocol for a single-blinded randomized sham-controlled clinical trial
Source: Trials. 2021 Sep 15;22:619. doi: 10.1186/s13063-021-05563-4 (PMC8441043; doi:10.1186/s13063-021-05563-4)
Supplement: Supplementary file 2 — Additional file 2. HAMD-17. [file 13063_2021_5563_MOESM2_ESM.docx]

**HAMD-17**

**1. DEPRESSED MOOD** (Sadness, hopeless, helpless, worthless)

**0=** Absent

**1=** These feeling states indicated only on questioning

**2=** These feeling states spontaneously reported verbally

**3=** Communicates feeling states non-verbally—i.e., through facial expression, posture, voice, and tendency to weep

**4=** Patient reports VIRTUALLY ONLY these feeling states in his spontaneous verbal and non verbal communication

**2. FEELINGS OF GUILT**

**0=** Absent

**1=** Self reproach, feels he has let people down

**2=** Ideas of guilt or rumination over past errors or sinful deeds

**3=** Present illness is a punishment. Delusions of guilt

**4=** Hears accusatory or denunciatory voices and/or experiences threatening visual hallucinations

**3. SUICIDE**

**0=** Absent

**1=** Feels life is not worth living

**2=** Wishes he were dead or any thoughts of possible death to self

**3=** Suicidal ideas or gesture

**4=** Attempts at suicide (any serious attempt rates 4)

**4. INSOMNIA EARLY**

**0=** No difficulty falling asleep

**1=** Complains of occasional difficulty falling asleep—i.e., more than 1/2 hour

**2=** Complains of nightly difficulty falling asleep

**5. INSOMNIA MIDDLE**

**0=** No difficulty

**1=** Patient complains of being restless and disturbed during the night

**2=** Waking during the night—any getting out of bed rates 2 (except for purposes of voiding)

**6. INSOMNIA LATE**

**0=** No difficulty

**1=** Waking in early hours of the morning but goes back to sleep

**2=** Unable to fall asleep again if he gets out of bed

**7. WORK AND ACTIVITIES**

**0=** No difficulty

**1=** Thoughts and feelings of incapacity, fatigue or weakness related to activities; work orhobbies

**2=** Loss of interest in activity; hobbies or work—either directly reported by patient, orindirect in listlessness, indecision and vacillation (feels he has to push self to work oractivities)

**3=** Decrease in actual time spent in activities or decrease in productivity

**4=** Stopped working because of present illness

**8. RETARDATION: PSYCHOMOTOR** (Slowness of thought and speech; impaired abilityto concentrate; decreased motor activity)

**0=** Normal speech and thought

**1=** Slight retardation at interview

**2=** Obvious retardation at interview

**3=** Interview difficult

**4=** Complete stupor

**9. AGITATION**

**0=** None

**1=** Fidgetiness

**2=** Playing with hands, hair, etc.

**3=** Moving about, can’t sit still

**4=** Hand wringing, nail biting, hair-pulling, biting of lips

**10. ANXIETY (PSYCHOLOGICAL)**

**0=** No difficulty

**1=** Subjective tension and irritability

**2=** Worrying about minor matters

**3=** Apprehensive attitude apparent in face or speech

**4=** Fears expressed without questioning

**11. ANXIETY SOMATIC:** Physiological concomitants of anxiety, (i.e., effects of autonomicoveractivity, “butterflies,” indigestion, stomach cramps, belching, diarrhea, palpitations,hyperventilation, paresthesia, sweating, flushing, tremor, headache, urinary frequency).Avoid asking about possible medication side effects (i.e., dry mouth, constipation)

**0=** Absent

**1=** Mild

**2=** Moderate

**3=** Severe

**4=** Incapacitating

**12. SOMATIC SYMPTOMS (GASTROINTESTINAL)**

**0=** None

**1=** Loss of appetite but eating without encouragement from others. Food intakeabout normal

**2=** Difficulty eating without urging from others. Marked reduction of appetite andfood intake

**13. SOMATIC SYMPTOMS GENERAL**

**0=** None

**1=** Heaviness in limbs, back or head. Backaches, headache, muscle aches. Loss of energyand fatigability

**2=** Any clear-cut symptom rates 2

**14. GENITAL SYMPTOMS** (Symptoms such as: loss of libido; impaired sexual performance;menstrual disturbances)

**0=** Absent

**1=** Mild

**2=** Severe

**15. HYPOCHONDRIASIS**

**0=** Not present

**1=** Self-absorption (bodily)

**2=** Preoccupation with health

**3=** Frequent complaints, requests for help, etc.

**4=** Hypochondriacal delusions

**16. LOSS OF WEIGHT**

**0=** No weight loss

**1=** Probably weight loss associated with present illness

**2=** Definite (according to patient) weight loss

**3=** Not assessed

**17. INSIGHT**

**0=** Acknowledges being depressed and ill

**1=** Acknowledges illness but attributes cause to bad food, climate, overwork, virus, needfor rest, etc.

**2=** Denies being ill at all
