# Supplementary material for: Electro-acupuncture for irritable bowel syndrome patients: study protocol for a single-blinded randomized sham-controlled clinical trial
Source: Trials. 2021 Sep 15;22:619. doi: 10.1186/s13063-021-05563-4 (PMC8441043; doi:10.1186/s13063-021-05563-4)
Supplement: Supplementary file 3 — Additional file 3. CGI-S. [file 13063_2021_5563_MOESM3_ESM.docx]

**CGI-S**

The CGI-S asks the clinician one question: “Considering your total clinical experience with this particular population, how mentally ill is the patient at this time?” which is rated on the following seven-point scale: 1=normal, not at all ill;2=borderline mentally ill; 3=mildly ill; 4=moderately ill; 5=markedly ill;6=severely ill; 7=among the most extremely ill patients

| 1 | Normal—not at all ill, symptoms of disorder not present past seven days |
| --- | --- |
| 2 | Borderline mentally ill—subtle or suspected pathology |
| 3 | Mildly ill—clearly established symptoms with minimal, if any, distress or difficulty in social and occupational function |
| 4 | Moderately ill—overt symptoms causing noticeable, but modest, functional impairment or distress; symptom level may warrant medication |
| 5 | Markedly ill—intrusive symptoms that distinctly impair social/occupational function or cause intrusive levels of distress |
| 6 | Severely ill—disruptive pathology, behavior and function are frequently influenced by symptoms, may require assistance from others |
| 7 | Among the most extremely ill patients—pathology drastically interferes in many life functions; may be hospitalized |
