# Supplementary material for: Electro-acupuncture for irritable bowel syndrome patients: study protocol for a single-blinded randomized sham-controlled clinical trial
Source: Trials. 2021 Sep 15;22:619. doi: 10.1186/s13063-021-05563-4 (PMC8441043; doi:10.1186/s13063-021-05563-4)
Supplement: Supplementary file 4 — Additional file 4. SDS. [file 13063_2021_5563_MOESM4_ESM.docx]

**SDS**

|  | A little of the time | Some of the time | Good part of the time | Most of the time |
| --- | --- | --- | --- | --- |
| 1. I feel down-hearted and blue | 1 | 2 | 3 | 4 |
| 2. Morning is when I feel the best | 4 | 3 | 2 | 1 |
| 3. I have crying spells or feel like it | 1 | 2 | 3 | 4 |
| 4. I have trouble sleeping at night | 1 | 2 | 3 | 4 |
| 5. I eat as much as I used to | 4 | 3 | 2 | 1 |
| 6. I still enjoy sex | 4 | 3 | 2 | 1 |
| 7. I notice that I am losing weight | 1 | 2 | 3 | 4 |
| 8. I have trouble with constipation | 1 | 2 | 3 | 4 |
| 9. My heart beats faster than usual | 1 | 2 | 3 | 4 |
| 10. I get tired for no reason | 1 | 2 | 3 | 4 |
| 11. My mind is as clear as it used to be | 4 | 3 | 2 | 1 |
| 12. I find it easy to do the things I used to | 4 | 3 | 2 | 1 |
| 13. I am restless and can’t keep still | 1 | 2 | 3 | 4 |
| 14. I feel hopeful about the future | 4 | 3 | 2 | 1 |
| 15. I am more irritable than usual | 1 | 2 | 3 | 4 |
| 16. I find it easy to make decisions | 4 | 3 | 2 | 1 |
| 17. I feel that I am useful and needed | 4 | 3 | 2 | 1 |
| 18. My life is pretty full | 4 | 3 | 2 | 1 |
| 19. I feel that others would be better off if I were dead | 1 | 2 | 3 | 4 |
| 20. I still enjoy the things I used to do | 4 | 3 | 2 | 1 |
