# Supplementary material for: Electro-acupuncture for irritable bowel syndrome patients: study protocol for a single-blinded randomized sham-controlled clinical trial
Source: Trials. 2021 Sep 15;22:619. doi: 10.1186/s13063-021-05563-4 (PMC8441043; doi:10.1186/s13063-021-05563-4)
Supplement: Supplementary file 5 — Additional file 5. IBS-QoL. [file 13063_2021_5563_MOESM5_ESM.docx]

**IBS-QoL**

| 1. I feel helpless because of my bowel problems. |
| --- |
| □_4_ Extremely □_3_Quite a bit □_2_Moderately □_1_Slightly □_0_Not at all |
| 2. I am embarrassed by the smell caused by my bowel problems. |
| □_4_ Extremely □_3_Quite a bit □_2_Moderately □_1_Slightly □_0_Not at all |
| 3. I am bothered by how much time I spend on the toilet. |
| □_4_A great deal □_3_Quite a bit □_2_Moderately □_1_Slightly □_0_Not at al |
| 4. I feel vulnerable to other illnesses because of my bowel problems. |
| □_4_ Extremely □_3_Quite a bit □_2_Moderately □_1_Slightly □_0_Not at all |
| 5. I feel fat because of my bowel problems. |
| □_4_A great deal □_3_Quite a bit □_2_Moderately □_1_Slightly □_0_Not at al |
| 6. I feel like I'm losing control of my life because of my bowel problems. |
| □_4_A great deal □_3_Quite a bit □_2_Moderately □_1_Slightly □_0_Not at al |
| 7. I feel my life is less enjoyable because of my bowel problems. |
| □_4_A great deal □_3_Quite a bit □_2_Moderately □_1_Slightly □_0_Not at al |
| 8. I feel uncomfortable when I talk about my bowel problems. |
| □_4_ Extremely □_3_Quite a bit □_2_Moderately □_1_Slightly □_0_Not at all |
| 9. I feel depressed about my bowel problems. |
| □_4_ Extremely □_3_Quite a bit □_2_Moderately □_1_Slightly □_0_Not at all |
| 10. I feel isolated from others because of my bowel problems. |
| □_4_ Extremely □_3_Quite a bit □_2_Moderately □_1_Slightly □_0_Not at all |
| 11. I have to watch the amount of food I eat because of my bowel problems. |
| □_4_A great deal □_3_Quite a bit □_2_Moderately □_1_Slightly □_0_Not at al |
| 12. Because of my bowel problems, sexual activity is difficult for me. |
| □_4_ Extremely □_3_Quite a bit □_2_Moderately □_1_Slightly □_0_Not at all |
| 13. I feel angry that I have bowel problems. |
| □_4_ Extremely □_3_Quite a bit □_2_Moderately □_1_Slightly □_0_Not at all |
| 14. I feel like I irritate others because of my bowel problems. |
| □_4_A great deal □_3_Quite a bit □_2_Moderately □_1_Slightly □_0_Not at al |
| 15. I worry that my bowel problems will get worse. |
| □_4_A great deal □_3_Quite a bit □_2_Moderately □_1_Slightly □_0_Not at al |
| 16. I feel irritable because of my bowel problems. |
| □_4_ Extremely □_3_Quite a bit □_2_Moderately □_1_Slightly □_0_Not at all |
| 17. I worry that people think I exaggerate my bowel problems. |
| □_4_A great deal □_3_Quite a bit □_2_Moderately □_1_Slightly □_0_Not at al |
| 18. I feel I get less done because of my bowel problems. |
| □_4_A great deal □_3_Quite a bit □_2_Moderately □_1_Slightly □_0_Not at al |
| 19. I have to avoid stressful situations because of my bowel problems. |
| □_4_A great deal □_3_Quite a bit □_2_Moderately □_1_Slightly □_0_Not at al |
| 20. My bowel problems reduce my sexual desire. |
| □_4_A great deal □_3_Quite a bit □_2_Moderately □_1_Slightly □_0_Not at al |
| 21. My bowel problems limit what I can wear. |
| □_4_A great deal □_3_Quite a bit □_2_Moderately □_1_Slightly □_0_Not at al |
| 22. I have to avoid strenuous activity because of my bowel problems. |
| □_4_A great deal □_3_Quite a bit □_2_Moderately □_1_Slightly □_0_Not at al |
| 23. I have to watch the kind of food I eat because of my bowel problems. |
| □_4_A great deal □_3_Quite a bit □_2_Moderately □_1_Slightly □_0_Not at al |
| 24. Because of my bowel problems, I have difficulty being around people I do not know well. |
| □_4_A great deal □_3_Quite a bit □_2_Moderately □_1_Slightly □_0_Not at al |
| 25. I feel sluggish because of my bowel problems. |
| □_4_ Extremely □_3_Quite a bit □_2_Moderately □_1_Slightly □_0_Not at all |
| 26. I feel unclean because of my bowel problems. |
| □_4_ Extremely □_3_Quite a bit □_2_Moderately □_1_Slightly □_0_Not at all |
| 27. Long trips are difficult for me because of my bowel problems. |
| □_4_ Extremely □_3_Quite a bit □_2_Moderately □_1_Slightly □_0_Not at all |
| 28. I feel frustrated that I cannot eat when I want because of my bowel problems. |
| □_4_ Extremely □_3_Quite a bit □_2_Moderately □_1_Slightly □_0_Not at all |
| 29. It is important to be near a toilet because of my bowel problems. |
| □_4_ Extremely □_3_Quite a bit □_2_Moderately □_1_Slightly □_0_Not at all |
| 30. My life revolves around my bowel problems. |
| □_4_A great deal □_3_Quite a bit □_2_Moderately □_1_Slightly □_0_Not at al |
| 31. I worry about losing control of my bowels in recent one week. |
| □_4_A great deal □_3_Quite a bit □_2_Moderately □_1_Slightly □_0_Not at al |
| 32. I fear that I won't be able to have a bowel movement in recent one week. |
| □_4_A great deal □_3_Quite a bit □_2_Moderately □_1_Slightly □_0_Not at al |
| 33. My bowel problems are affecting my closest relationships in recent one week. |
| □_4_A great deal □_3_Quite a bit □_2_Moderately □_1_Slightly □_0_Not at al |
| 34. I feel that no one understands my bowel problems in recent one week. |
| □_4_ Extremely □_3_Quite a bit □_2_Moderately □_1_Slightly □_0_Not at all |
