# Supplementary material for: Electro-acupuncture for irritable bowel syndrome patients: study protocol for a single-blinded randomized sham-controlled clinical trial
Source: Trials. 2021 Sep 15;22:619. doi: 10.1186/s13063-021-05563-4 (PMC8441043; doi:10.1186/s13063-021-05563-4)
Supplement: Supplementary file 6 — Additional file 6. Consent form. [file 13063_2021_5563_MOESM6_ESM.docx]

**HONG KONG BAPTIST UNIVERSITY INFORMED CONSENT STATEMENT**

**Acupuncture for Irritable Bowel Syndrome Patients:**

**A Single-blinded Randomized Sham-controlled Clinical Trial**

You are invited to participate in a research study. The purpose of this study is to determine whether acupuncture could have significant benefits than sham acupuncture for symptoms in IBS patients

**Background**

Irritable bowel syndrome (IBS) is one of the most commonly functional gastrointestinal disorders in clinical practice. To date, the treatment for IBS is unsatisfactory. Moreover, people with IBS frequently suffer from anxiety and depression, which can worsen symptoms. Failing to receive satisfactory treatment from western medicine, many IBS sufferers turned to Chinese medicine treatment, including acupuncture treatment.

**Study Aim**

The aim of this clinical study is to determine whether acupuncture could have significant benefits than sham acupuncture for symptoms in IBS patients.

**Project Title**

Acupuncture for Irritable bowel syndrome patients: a single-blinded randomized sham controlled clinical trial

**Study Plan**

This is a single blinded randomized sham controlled clinical trial with two arms. 120 IBS patients will be recruited. After a 2-week run-in period, eligible subjects will be randomly assigned to one of two arms, acupuncture (AC) arm and sham acupuncture (SAC) arm. Each eligible subject will go through a 2-wk run-in-period, 6-wk treatment period and 6-wk of follow-up period. Five visits in total are scheduled for each subject at week 0, week 2, week 5, week 8 and week 14. Totally there will be 12 sessions of acupuncture treatment, 2 sessions per week, and each session lasts 30 minutes.

**Risks and Emergency Medical Treatment**

Acupuncture treatment may cause the feeling of soreness, numbness, and heaviness, but, in general, does not cause significant discomfort and severe side effects. However, sometimes mild pain and bruise may occur in acupuncture points. Once any discomforts and unexpected symptoms and side effects occur, we will immediately notify you so that you could make decision whether you continue to participate in the study. Further medical treatment or referral to Mr. and Mrs. Chan Hon Yin Modern Chinese Medicine Research and Service Centre will be arranged, if necessary.

**Benefits**

Research leads to many advances in diagnosis and treatment of illness. Taking part in this research may not only benefit to you individually, but also helps to find a treatment for Irritable bowel syndrome. However, if positive results could be obtained from this study, your participation is no doubt beneficial to you and other patients in the future. Free charges for acupuncture treatment will be provided to you.

**Responsibilities**

If you agree to participate in this study, you need to provide your personal information, including name, age, address, telephone number, email address, and medical history.

**Confidentiality**

All information is only for research use and no personal information would be released. All the data will be destroyed after 5 years of the completion of the research study.

**Compensation and Insurance**

You have no compensation and charge for participating of this study. After the completion of this study, you can still seek for medical treatment in our clinics at your own cost. The study is covered by professional liability insurance policy.

**Contact**

If you have questions at any time about the study or the procedures, you may contact our research team.

Principle investigator: Dr. Linda Zhong

Telephone number: 3411 6523 Fax number: 3411 2929

If you feel you have not been treated according to the descriptions in this form, or your right as a participant in research have been violated during the course of this project, you may contact Research Ethics Committee, Hong Kong Baptist University(Contact Email: hkbu_rec@hkbu.edu.hk).

**Participation**

Your participant in this study is voluntary; you may decline to participate without penalty. If you decide to participate, you may withdraw from the study at any time without penalty and without loss of benefits to which you are otherwise entitled. If you withdraw from the study before data collection is completed your data will not be returned to you or destroyed.

**Consent**

I have read and understand the above information. I have received a copy of this form. I agree to participate in this study.

Subject's signature Date

Investigator's signature Date
