# Supplementary material for: Electro-acupuncture for irritable bowel syndrome patients: study protocol for a single-blinded randomized sham-controlled clinical trial
Source: Trials. 2021 Sep 15;22:619. doi: 10.1186/s13063-021-05563-4 (PMC8441043; doi:10.1186/s13063-021-05563-4)
Supplement: Supplementary file 7 — Additional file 7. Questionnaire with the Bristol Stool Form Scale. [file 13063_2021_5563_MOESM7_ESM.docx]

The Bristol Stool Form Scale (BSFS) is used to record stool consistency on days when patients are experiencing abnormal bowel habits and defines the following 7 types:

Type 1: separate hard lumps, like nuts; hard to pass

Type 2: sausage-shaped but lumpy

Type 3: like a sausage but with cracks on the surface

Type 4: like a sausage or snake, smooth and soft

Type 5: soft blobs with clear-cut edges

Type 6: fluffy pieces with ragged edges, a mushy stool

Type 7: watery, no solid pieces, entirely liquid

The IBS subtypes comprise 3 classifications based on the predominant bowel disorder and include IBS-D, IBS with constipation (IBS-C), and IBS with mixed symptoms of constipation and diarrhea (IBS-M). Individuals with a diagnosis of IBS whose bowel habits cannot be classified as IBS-D, IBS-C, or IBS-M are considered to have unclassified IBS (IBS-U).

IBS-D: More than 25% of bowel movements using the BSFS are type 6 or 7, and less than 25% of bowel movements are type 1 or 2.

IBS-C: More than 25% of bowel movements are type 1 or 2 using the BSFS, and less than 25% are type 6 or 7.

IBS-M: More than 25% of bowel movements using the BSFS are types 1 and 2, and more than 25% are types 6 and 7.

Patients who have the IBS-U subtype meet the diagnostic requirement for IBS, but their bowel habits cannot be accurately categorized as IBS-D, IBS-C, or IBS-M.
